# Probabilistic Methods for Verbal Autopsy Interpretation: InterVA Robustness in Relation to Variations in *A Priori* Probabilities

**DOI:** 10.1371/journal.pone.0027200

**Published:** 2011-11-03

**Authors:** Edward Fottrell, Kathleen Kahn, Stephen Tollman, Peter Byass

**Affiliations:** 1 Department of Public Health and Clinical Medicine, Umeå Centre for Global Health Research, Umeå University, Umeå, Sweden; 2 Centre for International Health and Development, Institute of Child Health, University College London, London, United Kingdom; 3 MRC/Wits Rural Public Health and Health Transitions Research Unit (Agincourt), School of Public Health, Faculty of Health Sciences, University of the Witwatersrand, Johannesburg, South Africa; Kenya Medical Research Institute - Wellcome Trust Research Programme, Kenya

## Abstract

**Background:**

InterVA is a probabilistic method for interpreting verbal autopsy (VA) data. It uses *a priori* approximations of probabilities relating to diseases and symptoms to calculate the probability of specific causes of death given reported symptoms recorded in a VA interview. The extent to which InterVA's ability to characterise a population's mortality composition might be sensitive to variations in these *a priori* probabilities was investigated.

**Methods:**

*A priori* InterVA probabilities were changed by 1, 2 or 3 steps on the logarithmic scale on which the original probabilities were based. These changes were made to a random selection of 25% and 50% of the original probabilities, giving six model variants. A random sample of 1,000 VAs from South Africa, were used as a basis for experimentation and were processed using the original InterVA model and 20 random instances of each of the six InterVA model variants. Rank order of cause of death and cause-specific mortality fractions (CSMFs) from the original InterVA model and the mean, maximum and minimum results from the 20 randomly modified InterVA models for each of the six variants were compared.

**Results:**

CSMFs were functionally similar between the original InterVA model and the models with modified *a priori* probabilities such that even the CSMFs based on the InterVA model with the greatest degree of variation in the *a priori* probabilities would not lead to substantially different public health conclusions. The rank order of causes were also similar between all versions of InterVA.

**Conclusion:**

InterVA is a robust model for interpreting VA data and even relatively large variations in *a priori* probabilities do not affect InterVA-derived results to a great degree. The original physician-derived *a priori* probabilities are likely to be sufficient for the global application of InterVA in settings without routine death certification.

## Introduction

Population-level cause-of-death data are a crucial component of understanding health and disease and formulating effective public health programs. Yet having one's death counted and assigned a cause, information critical to individual and population-level understandings of mortality, remains confined to a privileged minority of the world's population. For the majority of people in low-income settings who die at home without an attending physician and where deaths are not routinely recorded or classified by cause, verbal autopsy (VA) methods are the only viable means of deriving probable cause of death.

Though specific procedures can vary, VA is essentially the process of interviewing family, friends or carers after a death has occurred to find out about the circumstances of death. These data are normally gathered by lay interviewers and once gathered, the data are interpreted to derive possible cause(s) of death [1]. Physician review of VA data to assign a cause of death is historically the most commonly used method to derive possible causes, despite considerable concerns over the reliability and inefficient nature of this method. Recent advances in the development of computer-based probabilistic methods for interpreting VA are an attractive alternative to case-by-case physician interpretation. Such methods have the considerable advantage of being faster, cheaper and more internally consistent than physician review, offering new opportunities for timely and comparable cause-specific mortality estimates across time and space. High levels of agreement between probabilistic methods and physician review have been demonstrated in a number of different contexts and have highlighted important advantages of probabilistic methods [2]–[6].

InterVA is a widely used public-domain probabilistic method for interpreting VA data and has been applied in numerous settings in Africa and Asia [7]–[10]. InterVA is based on approximations of the probabilities of specific causes and specific symptoms among all deaths, as well as the probabilities of specific symptoms given that an individual has died from a specific cause. Using Bayes' theorem, it is then possible to calculate the probability of specific causes given reported symptoms recorded in a VA interview. In other words, InterVA obtains posterior probabilities of specific causes of death given an *a priori* distribution of the probabilities of causes and signs and symptoms of causes. Designed from the outset as a tool that can be applied in any setting where VAs are used, the *a priori* probabilities used to calculate the likelihoods of specific causes of death given a particular set of reported signs and symptoms are approximations that were agreed upon by a physician panel that included practitioners from a range of medical specialisations and geographic regions [2]. However, the extent to which InterVA's ability to characterise a population's mortality composition is sensitive to variations in these *a priori* probabilities, as might reflect regional differences in disease and symptom prevalences or indeed the opinions of a different panel of physicians, is unknown. Therefore, using a sample of deaths from the Agincourt Health and Socio-demographic Surveillance Site in rural South Africa as a test dataset for modelling, this study aims to test the sensitivity of InterVA to variations in the *a priori* probabilities that it uses. This has implications for the fundamental assumptions of InterVA and its use in a wide variety of settings with differing underlying mortality profiles. It is important to note that, as a methodological investigation, this paper does not set out to characterise the epidemiology or cause of death distribution of the Agincourt population.

## Methods

### InterVA

Applying Bayes' theorem, the computer-based InterVA approach calculates the probability of each of a finite list of causes (C) given the presence of specific signs, symptoms or indicators (I), for which the probability of reporting each indicator given a specific cause (P(I|C)) and the population-level probability of each cause among all deaths (P(C)) has been estimated [11]. In mathematical terms:
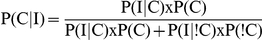
where P(!C) is the probability of not (C).

The prior probabilities P(I|C) and P(C) are derived from an expert physician consensus process whereby probabilities were estimated based on a range of thirteen approximate quantitative probabilities associated with semi-qualitative descriptors that included ‘impossible’ (P = 0), ‘uncommon’ (P = 0.001, P = 0.002 or P = 0.005), ‘moderately often’ (P = 0.01, P = 0.02 or P = 0.05), ‘frequently’ (P = 0.1, P = 0.2 or P = 0.5) and ‘inevitable’ (P = 1). Thus, each step increase on the scale results in an approximate doubling of the probability. The physicians involved in this process were selected from a range of settings and clinical backgrounds, thus minimising the risk of developing InterVA based too closely to the disease prevalence of any one geographical region or medical discipline [2].

Conceptually, InterVA is based on a matrix of *a priori* probabilities. The current InterVA model (version 3.2) is based on 35 possible causes of death, which can be considered as columns in the matrix, and 106 signs or symptoms (collectively referred to as ‘indicators’), which can be considered as rows in the matrix. The set of indicators and causes included in the model was influenced by established VA questionnaires and the expert consensus process described above, and can be viewed elsewhere [2] and online (www.interva.net). The matrix also includes one additional row and one additional column for the independent, baseline probabilities of causes (P(C)) and indicators (P(I)) among all deaths in a population. The cells in the matrix must then be populated with the probabilities of specific indicators given specific causes (P(I|C) (Table 1). As such the InterVA probability matrix is comprised of 3,852 cells (36 columns by 107 rows) each with a physician-consensus probability value.

**Table 1:** Illustration of the InterVA probability matrix of *a priori* probabilities.

			Causes
			C_1_		C_2_		...	C_36_	
			P(C_1_)		P(C_2_)		...	P(C_36_)	
	I_1_	P(I_1_)	P(I_1_|C_1_)		P(I_1_|C_2_)		...	P(I_1_|C_36_)	
	I_2_	P(I_2_)	P(I_2_|C_1_)		P(I_2_|C_2_)		...	P(I_2_|C_36_)	
Indicators	I_3_	P(I_3_)	P(I_3_|C_1_)		P(I_3_|C_2_)		...	P(I_3_|C_36_)	
	...	...	...		...		...	...	
	I_n_	P(I_106_)	P(I_106_|C_1_)		P(I_106_|C_2_)		...	P(I_106_|C_36_)	

Using the above equation, the probability of each cause can be determined based on the indicators reported in a VA. Symptoms, histories and circumstances of death reported in either the open narrative or closed questions in VA interviews can be utilised. InterVA displays up to three most likely causes of death with corresponding likelihoods and an overall certainty factor for each death. Fewer than three causes will be displayed if the probability of the third (or second) cause is less than 50% of the probability of the preceding cause. Cases with insufficient VA data to decisively determine the cause probabilities are identified by InterVA as ‘indeterminate’. Each assigned cause is associated with a likelihood, and the sum of likelihoods of assigned causes has a maximum value of 1.00. If the sum of likelihoods of assigned causes is less than 1.00, then the difference reflects a lack of certainty about the overall cause, which can be considered as an indeterminate component of the case [9].

### Random modifications to *a priori* probabilities

To assess the robustness of InterVA to variations in the *a priori* probabilities, routines were written using Microsoft Visual FoxPro software to select random samples of 25% and 50% of the matrix cells and change the *a priori* probabilities. Approximately half of the randomly selected cells had their probability increased and half had their probability decreased. Three different degrees of modification were used independently, i.e. up to 1, 2 and 3 steps on the logarithmic scale on which the original probabilities were based. Table 2 illustrates the procedure for each level of modification. No probabilities were made to equal 1 (inevitable) or 0 (impossible) and if probabilities could not be increased or decreased by the specified number of steps because they were already at or close to the maximum or minimum, they were increased or decreased by the maximum number of steps possible. If the original *a priori* probabilities could not increase or decrease at all, the direction of change was reversed. For example, a selected cell that already has a top probability (1.00 or 0.5) and is supposed to be increased by three steps will be decreased by 3 steps instead. This process was repeated twenty times for each degree of modification of the probability matrix thus generating 120 new matrices (i.e.20 matrices with 25% of the cells modified by 1 step, 20 matrices with 50% of the cells modified by 1 step, 20 matrices with 25% of the cells modified by 2 steps, and so on).

**Table 2:** Probability scale and qualitative descriptors on which *a priori* InterVA probabilities are based and a demonstration of how original probabilities were modified to varying degrees.

Qualitative Descriptor	Original Quantitative Probability	Increase by 1 Step	Decrease by 1 Step	Increase by 2 Steps	Decrease by 2 Step	Increase by 3 Steps	Decrease by 3 Step
Inevitable*	0.99	0.5α	0.5	0.2α	0.2	0.1α	0.1
Frequently	0.5	0.2α	0.2	0.1α	0.1	0.05α	0.05
	0.2	0.5	0.1	0.5γ	0.05	0.5γ	0.02
	0.1	0.2	0.05	0.5	0.02	0.5γ	0.01
Moderately Often	0.05	0.1	0.02	0.2	0.01	0.5	0.005
	0.02	0.05	0.01	0.1	0.005	0.2	0.002
	0.01	0.02	0.005	0.05	0.002	0.1	0.001
Uncommon	0.005	0.01	0.002	0.02	0.001	0.05	0.001δ
	0.002	0.005	0.001	0.01	0.001δ	0.02	0.001δ
	0.001	0.002	0.002β	0.005	0.005β	0.01	0.01β
Impossible*	0	0.001	0.001β	0.002	0.002β	0.005	0.005β

*No probabilities were made to be “inevitable” or “impossible”.

α Where probabilities could not be increased they were decreased by an equivalent amount.

β Where probabilities could not be decreased they were increased by an equivalent amount.

γ Where probabilities could not be increased by the full amount they were increased by as much as possible without violating *.

δ Where probabilities could not be decreased by the full amount they were decreased by as much as possible without violating *.

### The test dataset

The original and modified InterVA models were applied to a random sample of 1000 all-age, all-sex deaths occurring between 1992 and 2006 in the Agincourt Health and Socio-demographic Surveillance System (HDSS) in South Africa and for which VA data were available. The Agincourt HDSS is part of the INDEPTH network (www.indepth-network.org) and has monitored a contiguous population of around 70,000 since 1992. The background to this work as well as several cause-specific mortality analyses are described in detail elsewhere [9], [12]–[14]. The sample of 1,000 completed VAs represents approximately 17% of the available VA data, which is considered likely to provide an adequate representation of the range of VA-derived symptom profiles and major causes of death within this population.

### Sensitivity assessment

Population cause-specific mortality fractions (CSMFs) were derived for the same 1,000 VA cases for each randomised instance of the six InterVA model variants. To derive population level CSMFs, the sum of likelihoods for each cause category (including indeterminate) was divided by the sum of the likelihoods for all causes, thus splitting individual deaths between multiple causes weighted by the specific cause probabilities. The sum of all fractions in each cause category divided by the total number of deaths represents the population CSMF. The mean CSMFs and the rank order of causes from the 20 samples for each variation of the InterVA probability matrix were compared against each other and the results derived from the original, physician-consensus based model to assess functional (non-statistical) similarities.

### Ethical Approval

Within the Agincourt Health and Socio-demographic Surveillance project, informed consent is obtained verbally at the individual and household levels at every follow-up visit. Community consent from civic and traditional leadership was secured verbally at the start of surveillance and is reaffirmed during annual information feedback meetings. Written consent is not sought due to issues relating to literacy, the impracticalities of seeking repeated signed consent in a long running, prospective surveillance project, and a degree of local reluctance to signing forms, which relates to historical political experiences in South Africa's recent past. This process of informed consent and all surveillance-based studies in the Agincourt sub-district were reviewed and approved by the Committee for Research on Human Subjects (Medical) of the University of the Witwatersrand, Johannesburg, South Africa (protocol M960720).

## Results

Detailed CSMFs based on the original InterVA model using physician-derived *a priori* probabilities and the mean, minimum and maximum of twenty analyses of each of the modified probability model variants are shown in Table S1. Mean CSMFs were functionally similar between the original InterVA and each of the modified versions of it.

**Figure 1:**
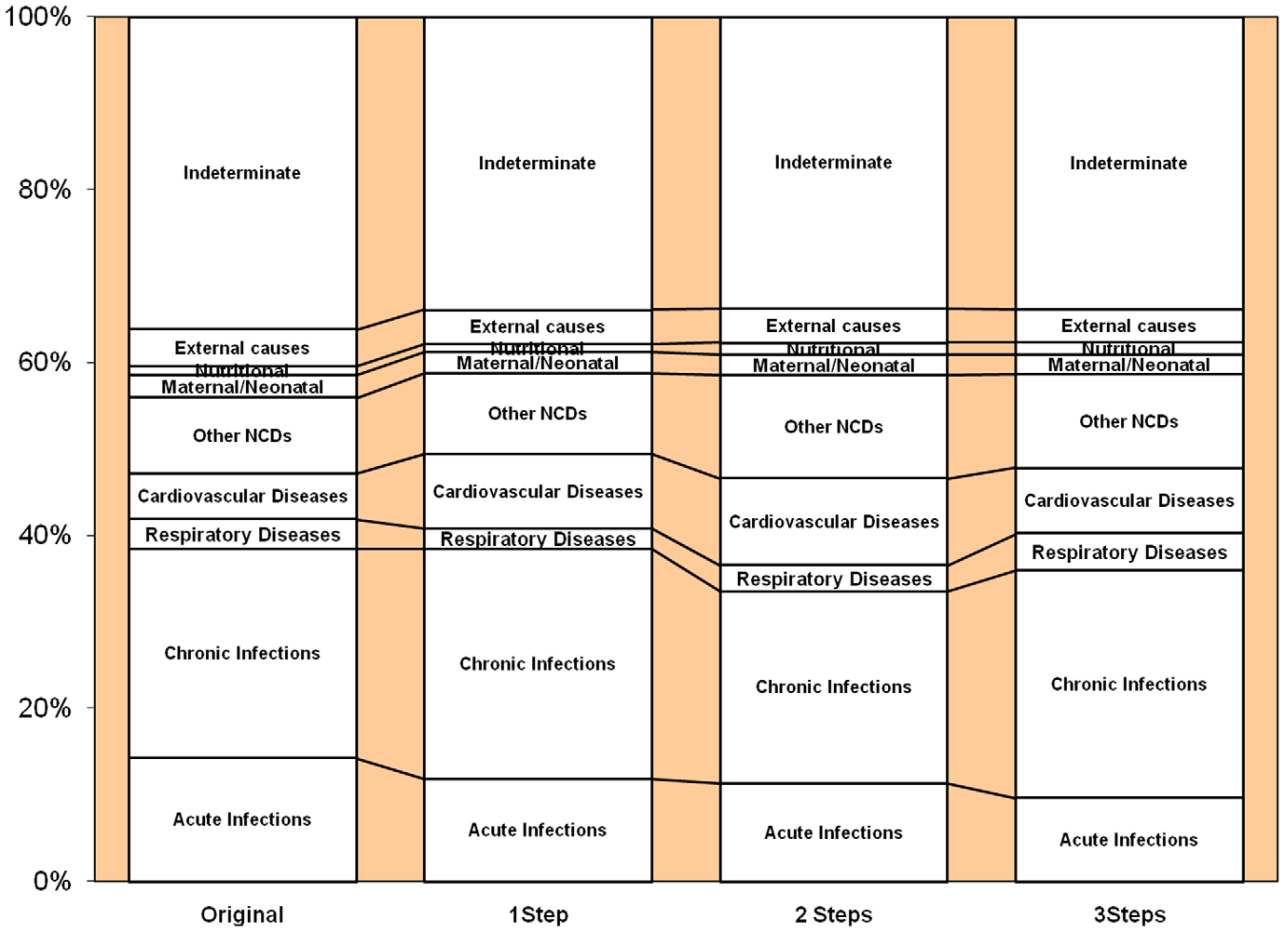
Broad cause distributions based on original InterVA and a modified version with 25% of *a priori* probabilities modified by upto 1, 2 and 3 steps.

**Figure 2:**
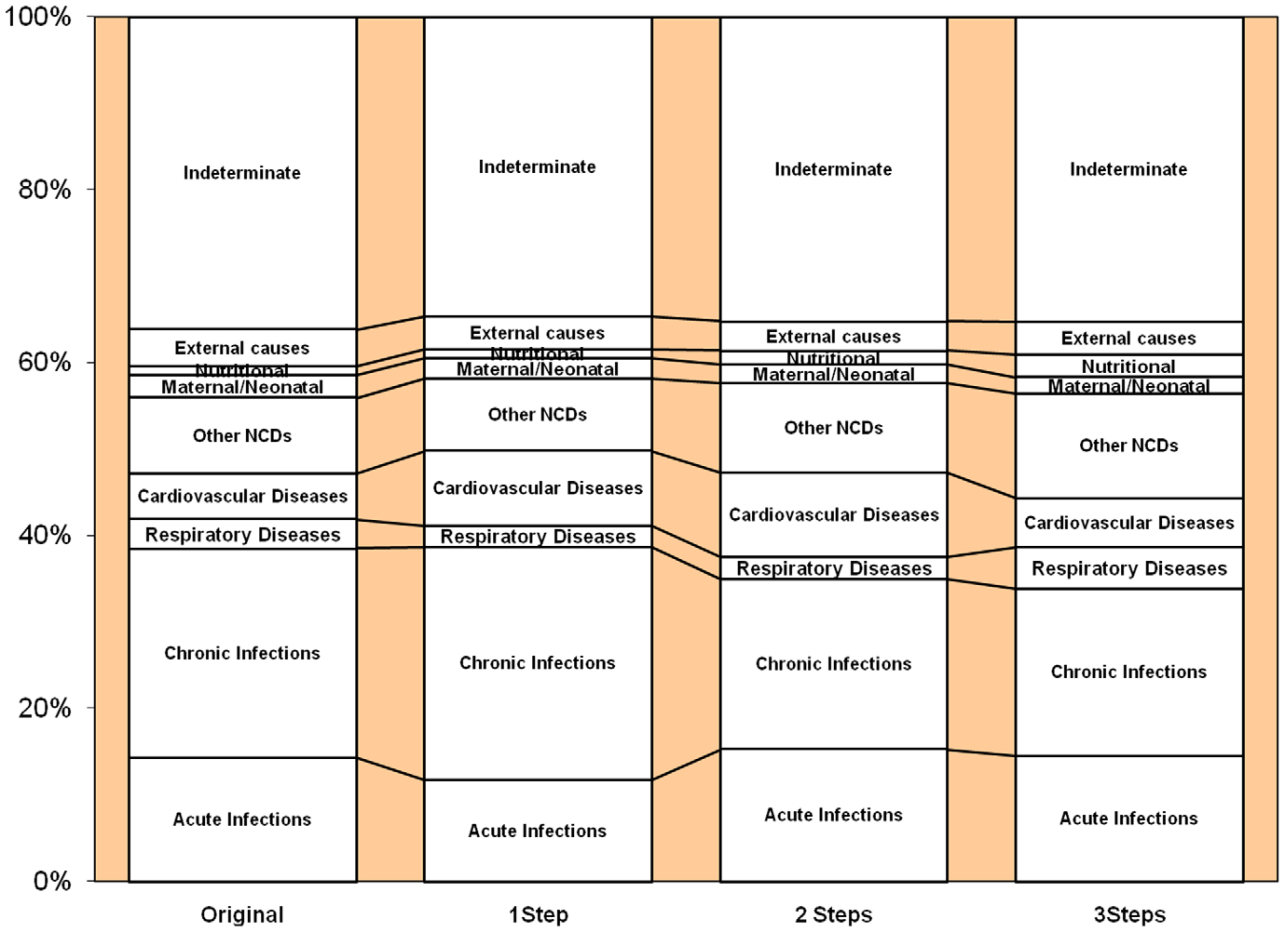
Broad cause distributions based on original InterVA and a modified version with 50% of *a priori* probabilities modified by upto 1, 2 and 3 steps.

When aggregated into broad cause categories, for which public health interventions and health service implications would not differ greatly, it was clear that any conclusions or understandings of cause-distributions based on any of the probability matrices would not differ greatly (Figures 1 & 2).

**Figure 3:**
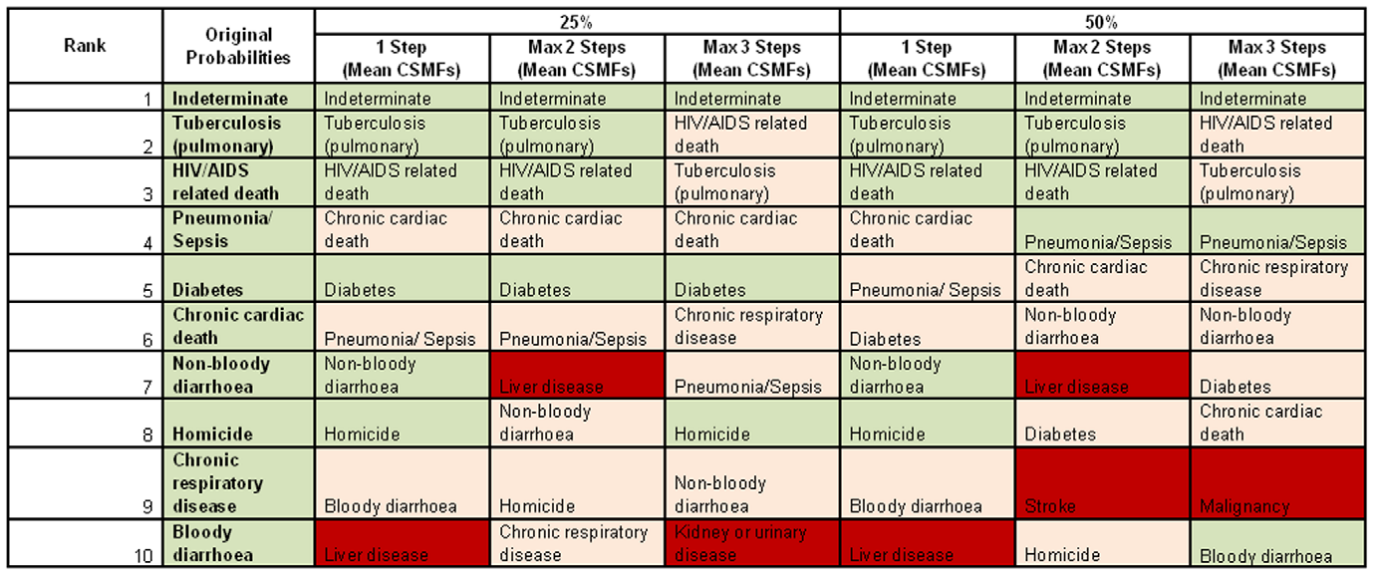
Top 10 cause-of-death categories based on InterVA and the physician-consensus *a priori* probabilities and the randomly modified probability matrices. Green shading represents an exact match, pink shading represents a top-10 cause but at a different rank, red shading represents a cause not included in the original top-10.

The rank order of the top ten causes of death according to each permutation of the probability matrix is shown in Figure 3. There was good overall agreement between all variations of InterVA; in all but one model, 9 out of 10 causes were common between the modified models and the original. The lowest level of agreement was for 50% variation at a maximum of 2 steps, but the agreement was still high, with 8 out of 10 causes common between the modified InterVA and the original.

## Discussion

From the outset, InterVA was designed to be a generic tool and not context-dependent. However, the extent to which a generic probability tool can be developed when underlying likelihoods of certain symptoms and causes inevitably vary across populations is yet to be established. With some important exceptions, large modifications to the *a priori* probabilities on which InterVA derives likely causes of death, resulting CSMFs, broad cause distributions and the rank order of causes did not generally differ to such an extent that one's overall impression of the major mortality burdens and public health priorities in the population would be likely to differ. This empirical study thus demonstrates a high degree of robustness of InterVA to variations in the *a priori* probability matrix, with important implications for the global application of the method.

Some important outliers were observed in the results. The maximum relative difference in a proportion attributed to a specific cause category compared to the original InterVA model was 215 times for malaria deaths (Figure 3, 50% of *a priori* probabilities changed by up to 3 steps). The extent to which such a large relative difference matter, however, depends on the original, or baseline, CSMF to which it relates and this 215-fold increase in the proportion of malaria deaths equates to an absolute increase of just over 2% compared to the original malaria mortality fraction. Conversely, absolute differences can be large and of public health importance if relative differences are small and the original CSMF is large. For example, the largest absolute difference observed was a 23% increase in the HIV-related mortality fraction (50% of *a priori* probabilities changed by up to 3 steps), representing an approximately 4-fold increase in this cause, with obvious public health implications. These results support the idea that it may be necessary to set InterVA *a priori* cause probabilities (P(C)) to account for local variations in cause prevalences only for those causes that are have a high baseline prevalence in some settings but a low prevalence in others.

Based on this reasoning the current InterVA model (version 3.2) expects inputs of “high” and “low” settings for HIV and malaria and adjusts the baseline probabilities accordingly. Although other causes of death included in InterVA are also likely to vary by at least an order of magnitude between populations, such as haemoglobinopathies or homicide, these causes are associated with such specific signs, symptoms and demographics that the need to allow for population differences is likely to be minimal.

This study limited the degree of variation in *a priori* probabilities at a maximum of three steps on the probability scale – equivalent to an order of magnitude. Though a greater degree of modification was possible, it is unlikely that any similarly diverse panel of physicians would reach conclusions on underlying population cause and indicator likelihoods that would differ by such a large degree, other than for HIV and malaria as described above. Given the findings that approximate, “ball-park” estimations of *a priori* probabilities are sufficient to achieve a workable model it is not surprising that the very first prototype of InterVA, which was based solely on the personal experiences and assumptions of an experienced epidemiologist, performed reasonably well [3]. Subsequent Delphi-style consensus building among a panel of physicians did improve the model's performance in relation to physician review [2]. However, observed improvements may have been as much due to the addition of new indicators and cause categories to the model as to modifications of *a priori* probabilities. Either way, any concerns that a different InterVA model would have resulted from the involvement of a different panel of physicians seem unfounded given the large degree of variation in underlying probabilities that is needed to meaningfully alter the output of InterVA.

The InterVA method does have limitations. For example, not all indicators built into InterVA are available in the Agincourt VA data and similarly, not all information gathered by the Agincourt VA questionnaire can be utilised by InterVA. In general the consequences of this are likely to lead to lower overall certainty of derived causes of death and the fact that InterVA cause probabilities are only modified by affirmative answers minimises the effects of missing or negative information from VA forms. In relation to the current study, it is possible that matrix probabilities that ultimately were not utilised when applied to the Agincourt data were included in the sample of modified probabilities. This is a limitation of the study and in theory could have underestimated the effect of *a priori* probability modification on resulting CSMFs. However, the repetition of each analysis twenty times, each time taking a new sample of matrix probability cells is likely to have reduced any such effects.

In presenting the considerable consistency of results from the InterVA model from these sensitivity analyses of randomly induced variations in the model's probability matrix, it is also important to stress that the model has previously been demonstrated to reveal very considerable differences in CSMFs across different settings in Africa and Asia [9]. It is therefore not the case that the InterVA model tends to reduce any VA data to a common pattern of causes of death, and thus the lack of variation in these sensitivity analyses does not arise out of an inherent property of the InterVA model.

Whilst this study shows that approximations of underlying probabilities in the InterVA model are sufficient to establish a workable model, the probabilities are based to some extent on the assumption that responses to each indicator are independent of all other indicators, which, in reality, is flawed. Other techniques are being developed that use facility-data to establish the probability of reporting specific symptoms given a specific cause [15], [16]. These symptom properties then allow population and individual-level cause patterns to be determined from VA data from a second dataset from the population of interest. Such methods are theoretically appealing but are ultimately limited in that they depend upon the availability of high quality facility-based or valid mortality data for modelling – a highly context-dependant pre-requisite that cannot readily be met in the majority of settings that need to use VA methods [1]. Furthermore, as the current study suggests, the added complexity and greater precision of such techniques may have little consequence on the ultimate conclusions and utility of VA-derived cause-of-death profiles.

VA is often considered a blunt tool that lacks precision. However, this need not detract from its utility for health monitoring and service planning in resource-poor settings that otherwise have no means of knowing and monitoring causes of death in their populations. Indeed, by keeping in mind who needs cause of death data and for what purposes [17], reasonable degrees of imprecision become acceptable and the criteria of efficiency, affordability and reliability become paramount. VA is fundamentally a population-level, public health tool; thus methods which reliably interpret VA data to estimate population-level causes of death with an appreciation for inevitable degrees of uncertainty are highly desirable and InterVA offers one such method that, based on this study's findings, can be applied with confidence in the diverse range of settings where deaths and their causes are not counted.

## Supporting Information

Table S1 **InterVA-derived cause-specific mortality fractions (%) of 1,000 deaths from Agincourt HDSS based on the original probability matrix and the mean, minimum and maximum CSMFs based on 20 randomly modified probability matrices in each of six model variants.**
(DOC)
