# Supplementary material for: Probabilistic Methods for Verbal Autopsy Interpretation: InterVA Robustness in Relation to Variations in A Priori Probabilities
Source: PLoS One. 2011 Nov 3;6(11):e27200. doi: 10.1371/journal.pone.0027200 (PMC3207846; doi:10.1371/journal.pone.0027200)
Supplement: Table S1 — InterVA-derived cause-specific mortality fractions (%) of 1,000 deaths from Agincourt HDSS based on the original probability matrix and the mean, minimum and maximum CSMFs based on 20 randomly modified probability matrices in each of six model variants. (DOC) [file pone.0027200.s001.doc]

| Cause | Original Probability  Matrix | 25% Random Sample Modified Variants | | | | | | | | | 50% Random Sample Modified Variants | | | | | | | | |
| --- | --- | --- | --- | --- | --- | --- | --- | --- | --- | --- | --- | --- | --- | --- | --- | --- | --- | --- | --- |
|  |  | Max 1 Step | | | Max 2 Steps | | | Max 3 Steps | | | Max 1 Step | | | Max 2 Steps | | | Max 3 Steps | | |
|  |  | Mean | Min | Max | Mean | Min | Max | Mean | Min | Max | Mean | Min | Max | Mean | Min | Max | Mean | Min | Max |
| Accidental drowning | 0.00 | 0.00 | 0.00 | 0.00 | 0.03 | 0.00 | 0.49 | 0.07 | 0.00 | 0.49 | 0.01 | 0.00 | 0.23 | 0.04 | 0.00 | 0.39 | 0.21 | 0.00 | 1.40 |
| Accidental poisoning | 0.08 | 0.14 | 0.06 | 0.30 | 0.13 | 0.00 | 0.28 | 0.41 | 0.00 | 1.47 | 0.11 | 0.00 | 0.23 | 0.48 | 0.03 | 1.59 | 0.53 | 0.00 | 2.68 |
| Acute cardiac death | 0.33 | 0.78 | 0.18 | 1.65 | 0.76 | 0.06 | 3.06 | 1.28 | 0.01 | 7.96 | 0.95 | 0.04 | 2.73 | 1.22 | 0.00 | 4.73 | 1.59 | 0.00 | 8.14 |
| Acute respiratory disease | 0.67 | 0.30 | 0.04 | 0.91 | 0.59 | 0.00 | 1.59 | 0.68 | 0.00 | 3.03 | 0.40 | 0.04 | 1.07 | 0.92 | 0.00 | 5.13 | 1.11 | 0.00 | 4.77 |
| Bloody diarrhoea | 2.34 | 2.24 | 1.53 | 3.33 | 2.25 | 0.08 | 4.25 | 1.91 | 0.43 | 4.14 | 2.42 | 1.09 | 4.30 | 1.64 | 0.01 | 5.00 | 2.45 | 0.02 | 10.32 |
| Chronic cardiac death | 4.49 | 7.06 | 1.17 | 14.56 | 8.28 | 1.22 | 18.37 | 5.17 | 0.82 | 21.57 | 6.77 | 1.42 | 17.01 | 6.15 | 0.65 | 19.13 | 2.80 | 0.03 | 8.86 |
| Chronic respiratory disease | 2.76 | 2.00 | 0.32 | 5.97 | 2.41 | 0.08 | 8.74 | 3.62 | 0.03 | 11.50 | 2.08 | 0.22 | 8.38 | 1.69 | 0.00 | 7.75 | 3.64 | 0.00 | 22.13 |
| Congenital malformation | 0.08 | 0.10 | 0.03 | 0.18 | 0.13 | 0.02 | 0.36 | 0.21 | 0.00 | 1.15 | 0.09 | 0.00 | 0.20 | 0.09 | 0.00 | 0.46 | 0.20 | 0.00 | 0.78 |
| Diabetes | 4.58 | 4.08 | 2.06 | 7.08 | 4.80 | 1.52 | 11.59 | 4.40 | 0.28 | 13.42 | 3.62 | 1.15 | 8.81 | 2.54 | 0.59 | 7.31 | 3.06 | 0.04 | 10.29 |
| Disease of nervous system | 0.00 | 0.02 | 0.00 | 0.11 | 0.06 | 0.00 | 0.38 | 0.14 | 0.00 | 1.16 | 0.02 | 0.00 | 0.18 | 0.13 | 0.00 | 0.90 | 0.68 | 0.00 | 2.96 |
| HIV/AIDS related death | 8.18 | 9.10 | 4.18 | 13.63 | 9.99 | 1.88 | 23.23 | 13.71 | 1.58 | 26.76 | 11.03 | 4.36 | 19.88 | 9.41 | 0.49 | 27.17 | 10.37 | 0.06 | 31.47 |
| Haemoglobinopathy | 0.19 | 0.14 | 0.00 | 0.32 | 0.11 | 0.00 | 0.67 | 0.19 | 0.00 | 0.72 | 0.11 | 0.00 | 0.35 | 0.36 | 0.00 | 2.86 | 0.67 | 0.00 | 3.94 |
| Homicide | 2.76 | 2.79 | 1.75 | 3.61 | 2.87 | 0.90 | 4.72 | 2.38 | 0.38 | 5.44 | 2.54 | 1.01 | 3.41 | 2.03 | 0.38 | 4.93 | 1.50 | 0.00 | 3.84 |
| Indeterminate | 36.13 | 33.89 | 30.41 | 36.92 | 33.71 | 28.82 | 37.58 | 33.82 | 29.19 | 40.08 | 34.67 | 30.18 | 37.87 | 35.25 | 30.56 | 40.74 | 35.30 | 31.79 | 41.23 |
| Kidney or urinary disease | 1.02 | 1.64 | 0.70 | 3.03 | 1.56 | 0.16 | 4.62 | 2.08 | 0.14 | 5.87 | 1.20 | 0.59 | 2.10 | 1.43 | 0.00 | 7.39 | 1.30 | 0.00 | 5.00 |
| Kwashiorkor | 0.00 | 0.04 | 0.00 | 0.28 | 0.04 | 0.00 | 0.18 | 0.06 | 0.00 | 0.30 | 0.03 | 0.00 | 0.16 | 0.10 | 0.00 | 1.06 | 0.12 | 0.00 | 0.64 |
| Liver disease | 2.27 | 2.15 | 0.99 | 5.35 | 3.53 | 0.08 | 9.49 | 1.45 | 0.02 | 8.28 | 2.10 | 0.64 | 5.94 | 2.94 | 0.08 | 10.82 | 2.28 | 0.00 | 7.10 |
| Malaria | 0.01 | 0.08 | 0.00 | 0.38 | 0.23 | 0.00 | 0.96 | 0.26 | 0.00 | 2.02 | 0.13 | 0.00 | 0.92 | 0.32 | 0.00 | 1.38 | 0.33 | 0.00 | 2.15 |
| Malignancy | 0.61 | 0.39 | 0.02 | 1.68 | 1.05 | 0.00 | 3.91 | 1.36 | 0.00 | 7.97 | 0.84 | 0.03 | 2.85 | 1.55 | 0.02 | 16.32 | 2.69 | 0.00 | 13.00 |
| Malnutrition | 1.04 | 0.86 | 0.20 | 2.46 | 1.38 | 0.18 | 4.72 | 1.41 | 0.03 | 6.72 | 1.02 | 0.03 | 2.77 | 1.51 | 0.02 | 5.06 | 2.44 | 0.02 | 15.13 |
| Maternity related death | 0.71 | 0.63 | 0.44 | 0.84 | 0.55 | 0.23 | 1.05 | 0.50 | 0.18 | 1.10 | 0.61 | 0.35 | 0.86 | 0.57 | 0.23 | 1.75 | 0.37 | 0.09 | 0.95 |
| Measles | 0.00 | 0.02 | 0.00 | 0.13 | 0.16 | 0.00 | 1.14 | 0.26 | 0.00 | 1.14 | 0.03 | 0.00 | 0.13 | 0.22 | 0.00 | 3.48 | 0.29 | 0.00 | 1.21 |
| Meningitis | 2.29 | 1.86 | 0.78 | 2.42 | 1.72 | 0.77 | 2.83 | 1.38 | 0.31 | 2.77 | 1.78 | 1.24 | 2.31 | 1.77 | 0.10 | 4.54 | 1.59 | 0.08 | 6.27 |
| Non-bloody diarrhoea | 4.10 | 3.55 | 1.45 | 5.95 | 2.92 | 0.43 | 8.13 | 2.11 | 0.07 | 6.75 | 3.32 | 1.69 | 7.16 | 3.84 | 0.35 | 14.54 | 3.19 | 0.00 | 11.35 |
| Other acute infection | 0.00 | 0.01 | 0.00 | 0.08 | 0.03 | 0.00 | 0.12 | 0.24 | 0.00 | 1.53 | 0.01 | 0.00 | 0.06 | 0.13 | 0.00 | 0.74 | 0.38 | 0.00 | 4.35 |
| Other chronic infection | 0.00 | 0.02 | 0.00 | 0.17 | 0.11 | 0.00 | 0.82 | 0.16 | 0.00 | 1.01 | 0.05 | 0.00 | 0.33 | 0.12 | 0.00 | 0.89 | 0.53 | 0.00 | 3.80 |
| Other digestive disease | 0.10 | 0.97 | 0.04 | 1.94 | 0.87 | 0.02 | 2.77 | 1.22 | 0.00 | 4.96 | 0.51 | 0.00 | 1.35 | 1.42 | 0.09 | 4.59 | 1.41 | 0.00 | 18.04 |
| Other fatal accident | 0.02 | 0.08 | 0.00 | 0.22 | 0.09 | 0.00 | 0.50 | 0.12 | 0.00 | 0.49 | 0.08 | 0.00 | 0.25 | 0.26 | 0.00 | 1.72 | 0.46 | 0.00 | 2.53 |
| Perinatal asphyxia | 1.22 | 1.13 | 0.92 | 1.46 | 0.99 | 0.24 | 1.49 | 1.04 | 0.28 | 1.72 | 1.01 | 0.37 | 1.58 | 0.83 | 0.23 | 1.40 | 0.70 | 0.00 | 1.81 |
| Pneumonia/Sepsis | 5.26 | 3.74 | 0.34 | 6.97 | 3.54 | 0.71 | 11.80 | 2.81 | 0.13 | 6.98 | 3.71 | 0.72 | 8.98 | 6.90 | 0.70 | 26.13 | 5.22 | 0.00 | 28.36 |
| Pre-term/small baby | 0.60 | 0.59 | 0.23 | 0.92 | 0.60 | 0.14 | 1.38 | 0.50 | 0.02 | 1.71 | 0.61 | 0.17 | 1.42 | 0.61 | 0.04 | 1.95 | 0.64 | 0.00 | 1.84 |
| Stroke | 0.46 | 0.81 | 0.29 | 1.56 | 1.02 | 0.03 | 3.59 | 1.12 | 0.00 | 3.60 | 0.94 | 0.09 | 5.11 | 2.38 | 0.03 | 10.64 | 1.34 | 0.00 | 6.96 |
| Suicide | 0.07 | 0.09 | 0.05 | 0.40 | 0.10 | 0.02 | 0.63 | 0.21 | 0.00 | 2.37 | 0.06 | 0.03 | 0.09 | 0.09 | 0.00 | 0.51 | 0.16 | 0.00 | 0.84 |
| Tetanus | 0.29 | 0.38 | 0.16 | 1.02 | 0.47 | 0.00 | 1.38 | 0.68 | 0.00 | 2.53 | 0.37 | 0.10 | 0.76 | 0.49 | 0.00 | 2.81 | 1.11 | 0.00 | 4.90 |
| Transport-related accident | 1.35 | 0.84 | 0.10 | 1.93 | 0.77 | 0.06 | 3.12 | 0.59 | 0.03 | 1.70 | 0.95 | 0.12 | 1.78 | 0.48 | 0.02 | 1.74 | 0.95 | 0.00 | 6.48 |
| Tuberculosis (pulmonary) | 16.00 | 17.49 | 10.20 | 29.37 | 12.12 | 1.08 | 27.97 | 12.42 | 3.17 | 25.26 | 15.82 | 7.30 | 36.74 | 10.11 | 1.03 | 32.11 | 8.40 | 0.28 | 30.63 |
